# Supplementary material for: Study on overburden failure law and surrounding rock deformation control technology of mining through fault
Source: PLoS One. 2022 Jan 24;17(1):e0262243. doi: 10.1371/journal.pone.0262243 (PMC8786183; doi:10.1371/journal.pone.0262243)
Supplement: S1 Data — (DOCX) [file pone.0262243.s001.docx]

**S1 Data. Normal excavation**

| Excavation distance cm | No.4 125cm | No.3 105cm | No.2 85cm | No.1 65cm |
| --- | --- | --- | --- | --- |
| 0 | 0 | 0 | 0 | 0 |
| 10 | 1E-4 | -1E-4 | 8E-4 | 7E-4 |
| 15 | -1E-4 | -1E-4 | 8E-4 | 7E-4 |
| 20 | -8E-4 | -1E-4 | 9E-4 | 9E-4 |
| 25 | -8E-4 | -1E-4 | 9E-4 | 0.001 |
| 30 | -6E-4 | 0 | 0.001 | 0.0014 |
| 35 | -7E-4 | 1E-4 | 0.0013 | 0.0016 |
| 40 | -8E-4 | 4E-4 | 0.002 | 0.0024 |
| 45 | -7E-4 | 9E-4 | 0.0025 | 0.004 |
| 50 | -6E-4 | 0.0016 | 0.0034 | 0.0079 |
| 55 | -8E-4 | 0.0019 | 0.0043 | 0.0148 |
| 60 | -3E-4 | 0.0019 | 0.0053 | 0.0307 |
| 65 | -0.001 | 0.0032 | 0.0072 | -9.8E-4 |
| 70 | -0.0011 | 0.0035 | 0.0082 | -0.00119 |
| 75 | -0.002 | 0.0063 | 0.0157 | -0.00105 |
| 80 | -0.0022 | 0.0082 | 0.0324 | -0.00126 |
| 85 | -9E-4 | 0.0111 | -0.0014 | -9.8E-4 |
| 90 | -9E-4 | 0.011 | -0.0017 | -0.00105 |
| 95 | 0 | 0.0207 | -0.0015 | -0.00119 |
| 100 | 9E-4 | 0.0371 | -0.0018 | -0.00168 |
| 105 | 0.0031 | -0.0012 | -0.0014 | -0.00126 |
| 110 | 0.0085 | 0.0013 | -0.0015 | -9.1E-4 |
| 115 | 0.0078 | 0.0021 | -0.0017 | 2.8E-4 |
| 120 | 0.019 | 0.0019 | -0.0024 | 7.7E-4 |
| 125 | -0.0029 | 0.0014 | -0.0018 | 9.8E-4 |
| 130 | -0.0014 | -0.0013 | -0.0013 | 7E-4 |
| 135 | -5E-4 | -0.0018 | 4E-4 | 0.0021 |
| 140 | -7E-4 | -0.00169 | 0.0011 | 0.0028 |
| 145 | -5E-4 | -0.002 | 0.0014 | 0.0028 |
| 150 | -8E-4 | -0.0019 | 0.001 | 0.0028 |
| 155 | -9E-4 | -0.003 | 0.003 | 0.00273 |
| 160 | 0.0013 | -0.0035 | 0.004 | 0.00273 |
| 165 | 0.0019 | -0.0031 | 0.004 | 0.00224 |
| 170 | 0.0015 | -0.0023 | 0.004 | 0.00231 |
| 175 | 0.0017 | -0.0012 | 0.0039 | 0.00231 |
| 180 | 0.0021 | -0.0013 | 0.0039 | 0.00217 |
| 185 | 0.001 | -0.0011 | 0.0032 | 0.00189 |
| 190 | 0.001 | -7E-4 | 0.0033 | 0.00161 |
| 195 | 0.0012 | -4E-4 | 0.0033 | 0.00147 |
| 200 | 0.0016 | -0.0013 | 0.0031 | 0.00112 |
| 205 | 0.0014 | -0.0018 | 0.0027 | 0.00105 |
| 210 | 0.0015 | -0.0017 | 0.0023 | 0.0013 |
| 215 | 0.0013 | -0.0017 | 0.0021 | 0.00125 |
| 220 | 0.0011 | -0.0019 | 0.0016 | 0.0013 |
| 225 | 0.0012 | -0.0022 | 0.0015 | 0.00117 |
