# Supplementary material for: Study on overburden failure law and surrounding rock deformation control technology of mining through fault
Source: PLoS One. 2022 Jan 24;17(1):e0262243. doi: 10.1371/journal.pone.0262243 (PMC8786183; doi:10.1371/journal.pone.0262243)
Supplement: S2 Data — (DOCX) [file pone.0262243.s002.docx]

**S2 Data. Through-the-fault excavation**

| Excavation distance cm | No.8 225cm | No.7 205cm | No.6 185cm | No.5 145cm |
| --- | --- | --- | --- | --- |
| 0 | 0 | 0 | 0 | 0 |
| 10 | 0 | 6E-5 | 1E-4 | 0 |
| 15 | 0 | 6E-5 | 4E-4 | 0 |
| 20 | -1E-4 | 6E-5 | 6E-4 | 1E-4 |
| 25 | 0 | 1.2E-4 | 5E-4 | -1E-4 |
| 30 | 1E-4 | 2.4E-4 | 4E-4 | -8E-4 |
| 35 | 4E-4 | 1.8E-4 | 5E-4 | -8E-4 |
| 40 | 6E-4 | 1.8E-4 | 6E-4 | -6E-4 |
| 45 | 6E-4 | 3.6E-4 | 9E-4 | -7E-4 |
| 50 | 0.0011 | 6E-5 | 3E-4 | -8E-4 |
| 55 | 0.0013 | 1.8E-4 | 2E-4 | -7E-4 |
| 60 | 0.0017 | 1.8E-4 | 2E-4 | -6E-4 |
| 65 | 0.0011 | 3.6E-4 | 1E-4 | -8E-4 |
| 70 | 9E-4 | 1.2E-4 | -2E-4 | -3E-4 |
| 75 | 8E-4 | 0.00162 | 0.0011 | -0.001 |
| 80 | 0.0012 | 0.00186 | 0.0019 | -0.0011 |
| 85 | 0.0016 | 0.00186 | 0.0024 | -0.002 |
| 90 | 0.0015 | 0.00234 | 0.0044 | -0.0022 |
| 95 | 0.0025 | 0.00228 | 0.0025 | -9E-4 |
| 100 | 0.0032 | 0.00354 | 0.005 | -9E-4 |
| 105 | 0.0037 | 0.00324 | 0.0028 | 0 |
| 110 | 0.0032 | 0.0027 | 0.0018 | 9E-4 |
| 115 | 0.0036 | 0.00342 | 0.0043 | 0.0031 |
| 120 | 0.0035 | 0.00324 | 0.0019 | 0.0085 |
| 125 | 0.0038 | 0.00372 | 0.0025 | 0.0078 |
| 130 | 0.0043 | 0.00396 | 0.0026 | 0.019 |
| 135 | 0.0044 | 0.00546 | 0.001 | -0.0029 |
| 140 | 0.0035 | 0.00402 | 2E-4 | -0.0014 |
| 145 | 0.0036 | 0.0048 | 3E-4 | -5E-4 |
| 150 | 0.0048 | 0.04914 | -0.0028 | -7E-4 |
| 155 | 0.0189 | 0.05526 | 0.0095 | -5E-4 |
| 160 | 0.0094 | 0.04206 | 0.0139 | -8E-4 |
| 165 | 0.0053 | 0.04146 | 0.0064 | -9E-4 |
| 170 | 0.0057 | 0.04062 | -0.0045 | 0.0013 |
| 175 | 0.0068 | 0.03594 | 0.0075 | 0.0019 |
| 180 | 0.0068 | 0.02232 | 0.01 | 0.0015 |
| 185 | 0.0127 | 0.01224 | 0.0326 | 0.0017 |
| 190 | 0.0123 | 0.01434 | 0.0356 | 0.0021 |
| 195 | 0.0167 | 0.01776 | 0.03 | 0.001 |
| 200 | 0.0223 | 0.04614 | 0.0272 | 0.001 |
| 205 | 0.0308 | 0.04356 | 0.0263 | 0.0012 |
| 210 | 0.0421 | 0.04158 | 0.0217 | 0.0016 |
| 215 | 0.0511 | 0.03822 | 0.0175 | 0.0014 |
| 220 | 0.0551 | 0.02952 | 0.005 | 0.0015 |
| 225 | 0.0055 | 0.0294 | 0.0054 | 0.0013 |
