# Supplementary material for: Study on overburden failure law and surrounding rock deformation control technology of mining through fault
Source: PLoS One. 2022 Jan 24;17(1):e0262243. doi: 10.1371/journal.pone.0262243 (PMC8786183; doi:10.1371/journal.pone.0262243)
Supplement: S3 Data — (DOCX) [file pone.0262243.s003.docx]

**S3 Data. The surrounding rock deformation-time diagram**

| Accumulated days/d | | A1 Level convergence /mm | A1 Siking vault /mm | A1 kick drum  /mm | Accumulated days/d | A2 Level convergence /mm | A2 Siking vault /mm | A2 kick drum  /mm | Accumulated days/d | A3 Level convergence /mm | A3 Siking vault /mm | A3 kick drum  /mm |
| --- | --- | --- | --- | --- | --- | --- | --- | --- | --- | --- | --- | --- |
| 1 | 3.9 | | 2.02 | 5.91 | 1 | 0.64 | 3.27 | 4.56 | 1 | 2.53 | 1.19 | 4.76 |
| 2 | 7.28 | | 4.41 | 10.38 | 2 | 2.21 | 3.76 | 7.03 | 2 | 3.1 | 1.69 | 8.23 |
| 3 | 10.64 | | 6.29 | 16.23 | 4 | 4.24 | 4.46 | 17.88 | 3 | 5.13 | 3.39 | 11.08 |
| 4 | 17.46 | | 10.15 | 24.06 | 5 | 4.42 | 5.21 | 24.71 | 4 | 6.31 | 4.34 | 12.91 |
| 5 | 23.14 | | 19.64 | 38.84 | 6 | 6.74 | 5.57 | 30.49 | 5 | 13.63 | 3.68 | 21.69 |
| 6 | 27.73 | | 22.04 | 51.02 | 7 | 8.45 | 5.59 | 38.3 | 6 | 17.34 | 5.3 | 19.5 |
| 8 | 37.42 | | 26.44 | 67.7 | 9 | 11.48 | 7.37 | 41.98 | 8 | 19.37 | 4.78 | 23.18 |
| 10 | 48.45 | | 29.37 | 65.86 | 11 | 19.07 | 11.78 | 45.82 | 10 | 20.96 | 5.19 | 33.02 |
| 12 | 60.66 | | 30.5 | 74.38 | 13 | 24.1 | 14.14 | 49.33 | 12 | 22.99 | 6.54 | 36.53 |
| 14 | 62.59 | | 37.97 | 90.95 | 17 | 29.83 | 18 | 59.91 | 14 | 25.72 | 6.41 | 41.11 |
| 15 | 68.34 | | 41.47 | 97.99 | 21 | 31.89 | 23.58 | 63.94 | 16 | 27.78 | 7.98 | 48.14 |
| 17 | 68.49 | | 41.65 | 100.98 | 24 | 38.21 | 23.61 | 70.94 | 19 | 30.1 | 9.02 | 53.14 |
| 19 | 71.99 | | 41.97 | 103.56 | 26 | 39.23 | 24.37 | 78.52 | 21 | 40.12 | 8.77 | 53.72 |
| 21 | 74.06 | | 43.75 | 106.5 | 29 | 41.4 | 29.75 | 81.45 | 24 | 42.29 | 9.39 | 57.65 |
| 23 | 76.12 | | 48.18 | 110.23 | 35 | 44.05 | 34.67 | 88.18 | 30 | 43.94 | 11.32 | 61.38 |
| 26 | 77.76 | | 49.46 | 118.18 | 38 | 45.64 | 36.54 | 90.14 | 34 | 46.53 | 10.49 | 68.34 |
| 29 | 79.8 | | 55.44 | 126.34 | 45 | 46.43 | 37.54 | 92.3 | 38 | 49.52 | 12.49 | 74.5 |
| 32 | 82.82 | | 57.95 | 127.69 | 46 | 46.63 | 38.52 | 94.64 | 41 | 52.52 | 14.47 | 76.84 |
| 36 | 85.15 | | 58.83 | 132.96 | 47 | 47.37 | 38.68 | 95.92 | 45 | 54.51 | 14.63 | 80.12 |
| 38 | 86.31 | | 62.05 | 133.12 | 52 | 49.1 | 40.75 | 98.37 | 50 | 56.99 | 16.7 | 80.57 |
| 42 | 88.43 | | 63.31 | 139.67 | 56 | 49.33 | 40.79 | 99.74 | 55 | 57.22 | 17.74 | 81.94 |
| 47 | 89.51 | | 63.52 | 142.04 | 66 | 49.58 | 41.83 | 101.65 | 59 | 59.27 | 18.78 | 85.85 |
| 52 | 90.03 | | 63.73 | 143.94 | 72 | 50.44 | 42.04 | 103.26 | 63 | 60.33 | 19.99 | 89.46 |
| 59 | 91.28 | | 64.43 | 145.55 |  |  |  |  | 67 | 63.03 | 19.92 | 90.94 |
| 62 | 92.11 | | 64.65 | 147.03 |  |  |  |  | 71 | 64.43 | 20.48 | 91.89 |
