# Supplementary material for: Study on overburden failure law and surrounding rock deformation control technology of mining through fault
Source: PLoS One. 2022 Jan 24;17(1):e0262243. doi: 10.1371/journal.pone.0262243 (PMC8786183; doi:10.1371/journal.pone.0262243)
Supplement: Attachment — Submitted filename: Responses to reviewers.docx [file pone.0262243.s005.docx]

**Responses to reviewers**

**Dear Editors and Reviewers,**

Thank you very much for your careful review of our paper. Those comments are all valuable and very helpful for revising and improving our paper, as well as the important guiding significance to our researches. We have revised the manuscript in response to your suggestions and questions. All the modifications made according to reviewer’s comments are highlighted in yellow and using track changes in the manuscript. Our responses are also outlined below following your comments. I hope the revised manuscript is acceptable for publication in our journal.

Sincerely,

Yuejin Zhou, Ph. D.

Corresponding author for the manuscript

**Ref: PONE-D-21-26499R1**

**Title: Study on overburden failure law and surrounding rock deformation control technology of mining through fault**

**Journal: PLOS ONE**

**Reviewer #1:**

1. Please continuously clarify the novelty of the MS because the MS does not well show the novelty.

Answer: Thanks for your comment. Aiming at the influence of mining face excavation on the roadway through fault, the geological strength index (GSI) was introduced to obtain the basis for judging the instability of the surrounding rock of the roadway through fault, and combined with two different similar tests to analyze the scope of roadway instability and the failure law of overlying rock masses. According to the conclusions obtained, the roadway support scheme is designed. This study shows that: 1) Through the introduction of GSI, it is found that the instability range of roadway surrounding rock is exponentially correlated with the rupture degree of surrounding rock. On this basis, the critical instability range of roadway surrounding rock is deduced to be 2.32m. 2) Compared with ordinary mining, through-the-fault mining causes slippage and dislocation of the fault, the load of the overburden is transferred to both sides of the fault, and the stress near the fault accumulates abnormally. The “three zones” characteristics of the overburden movement basically disappear, the subsidence pattern is changed from "trapezoid" to "inverted triangle", and the influence distance of the advance mining stress on the working face is extended from 20m to 30m. 3) According to the conclusion, the bolt length and roadway reinforced support length are redesigned. Engineering application shows that the deformation rate of the roadway within 60 days is controlled below 0.1~0.5mm/d, the deformation amount is controlled within 150mm, and the roadway deformation is controlled, which generally meets the requirements of use. The research results provide guidance and reference for similar roadway support design.

2. please check the reliability of result of 2.32m. In eq.20, the values of s shows 0.1 and 0.01, please check it.

Answer: Thank you very much for your careful review of our paper. The value of s is 0.01, we are very sorry that we missed the number when editing the formula. The overall calculation result is correct. The specific calculation process is as follows:

$$\begin{matrix} t=\frac{-m_{b}\sigma_{ci}+\sqrt{m_{b}^{2}\sigma_{ci}^{2}+16\sigma_{ci}^{2}s+16m_{b}\sigma_{ci}P}-4\sigma_{ci}\sqrt{s}}{2\sigma_{ci}m_{b}} \\ =\frac{-1.10\times16+\sqrt{1.10^{2}\times16^{2}+16\times16^{2}\times0.01+16\times1.10\times16\times9}-4\times16\sqrt{0.01}}{2\times16\times1.10} \\ \text{=}0.8441 \\ R=e^{t}\text{=}e^{0.8441}=2.32m \end{matrix}$$

3. It is suggested to remove the author marks of equally contuibution.

Answer: Thank you for this suggestion. We have removed the author marks of equally contuibution.

**Reviewer #2:**

1. What's the groundwater impact the fault movement? does it influnce your results?

Answer: Thanks for your comment. This paper studies the impact of mining face excavation on the roadway through fault. The activation of faults is mainly caused by mining face excavation, and the influence of groundwater in this process is small and does not influnce the results.
