# Supplementary material for: Study on overburden failure law and surrounding rock deformation control technology of mining through fault
Source: PLoS One. 2022 Jan 24;17(1):e0262243. doi: 10.1371/journal.pone.0262243 (PMC8786183; doi:10.1371/journal.pone.0262243)
Supplement: Attachment — Submitted filename: Responses to reviewers.docx [file pone.0262243.s006.docx]

**Responses to reviewers**

**Dear** **Editors and Reviewers,**

Thank you very much for your reminder, and thank you very much for your careful review of our paper. Those comments are all valuable and very helpful for revising and improving our paper, as well as the important guiding significance to our researches. We have revised the manuscript in response to your suggestions and questions. All the modifications made according to the reviewer’s comments are highlighted in yellow and using track changes in the manuscript. Our responses are also outlined below following your comments. I hope the revised manuscript is acceptable for publication in our journal. Finally, thank you again for your reminders and comments.

Sincerely,

Yuejin Zhou, Ph. D.

Corresponding author for the manuscript

**Ref: PONE-D-21-26499**

**Title: Study on overburden failure law and surrounding rock deformation control technology of mining through fault**

**Journal: PLOS ONE**

**Reviewer #1:**

1. Please continuously clarify the novelty of the MS because the MS does not well show the novelty.

Answer: Thanks for your comment. Your comment is very helpful for revising and improving our paper. We have revised the abstract section to better express the novelty of the MS in lines 11-15 and line 22 of the revised manuscript. The revised summary section is as follows：

In the mining process of working face, the additional stress generated by the fault changes the law of roadway deformation and failure as well as the law of overburden failure. Aiming at the influence of the fault in the mining process of working face, this study introduced the geological strength index (GSI) to analyze the stress distribution in the elastic-plastic zone of the surrounding rock of the roadway. And similar experiments under different engineering backgrounds were combined to study the characteristics of overburden movement and stress evolution. Based on the conclusions obtained, the roadway support scheme was designed. This study shows that, compared with ordinary mining, through-the-fault mining causes slippage and dislocation of the fault, the load of the overburden is transferred to both sides of the fault, and the stress near the fault accumulates abnormally. The “three zones” characteristics of the overburden movement disappear, the subsidence pattern is changed from "trapezoid" to "inverted triangle", and the influence distance of the advanced mining stress on the working face is extended from 20m to 30m. The instability range of roadway surrounding rock is exponentially correlated with the rupture degree of the surrounding rock. Through the introduction of GSI, the critical instability range of roadway surrounding rock is deduced to be 2.32m. According to the conclusion, the bolt length and roadway reinforced support length are redesigned. Engineering application shows that the deformation rate of the roadway within 60 days is controlled below 0.1~0.5mm/d, the deformation amount is controlled within 150mm, and the roadway deformation is controlled, which generally meets the requirements of use. The research results provide guidance and reference for similar roadway support.

2. please check the reliability of result of 2.32m. In eq.20, the values of s shows 0.1 and 0.01, please check it.

Answer: Thank you very much for your careful review of our paper. The value of s is 0.01, we are very sorry that we missed the number when editing the formula. The overall calculation result is correct. The specific calculation process is as follows:

$$\begin{matrix} t=\frac{-m_{b}\sigma_{ci}+\sqrt{m_{b}^{2}\sigma_{ci}^{2}+16\sigma_{ci}^{2}s+16m_{b}\sigma_{ci}P}-4\sigma_{ci}\sqrt{s}}{2\sigma_{ci}m_{b}} \\ =\frac{-1.10\times16+\sqrt{1.10^{2}\times16^{2}+16\times16^{2}\times0.01+16\times1.10\times16\times9}-4\times16\sqrt{0.01}}{2\times16\times1.10} \\ \text{=}0.8441 \\ R=e^{t}\text{=}e^{0.8441}=2.32m \end{matrix}$$

3. It is suggested to remove the author marks of equally contuibution.

Answer: Thank you for this suggestion. After our discussion, we have removed the author marks of equally contuibution in line 3 of the revised manuscript.

**Reviewer #2:**

1. What's the groundwater impact the fault movement? does it influnce your results?

Answer: Thanks for your comment. Your comment is very valuable for revising our paper. Generally, when a fault zone connects to aquifers, groundwater will spread to the fault. The pressure of pore fluid in the fault zone will increase, and the friction strength of the fault will decrease, which will accelerate fault instability. For this study, since the fault has a small drop and does not connect to the aquifer, the excavation of the working face under the influence of the fault is the main reason for roadway instability, so the groundwater is not considered. We have added relevant explanations in lines 188-190 of the revised manuscript. The distribution law of fault stress under the influence of groundwater, involves water-sediment seepage, the hydration reaction of fault, and the impact on roadway instability. We are researching these aspects, and we have achieved some results so far. We hope to publish relevant research results on PLOS ONE in the future.
